# Altered mRNA Expression Related to the Apoptotic Effect of Three Xanthones on Human Melanoma SK-MEL-28 Cell Line

**DOI:** 10.1155/2013/715603

**Published:** 2013-09-23

**Authors:** Jing J. Wang, Wei Zhang, Barbara J. S. Sanderson

**Affiliations:** ^1^Department of Medical Biotechnology, Flinders Medical Sciences and Technology, School of Medicine, Faculty of Health Science, Flinders University, Level 4, Health Science Building, Registry Road, Bedford Park, Adelaide, SA 5042, Australia; ^2^Flinders Centre for Marine Bioproducts Development (FCMBD), Flinders University, Level 4, Health Science Building, Registry Road, Bedford Park, Adelaide, SA 5042, Australia

## Abstract

We previously demonstrated that **α**-mangostin, **γ**-mangostin, and 8-deoxygartanin have significant cytotoxic effects on human melanoma SK-MEL-28 cell line. The current study revealed the underlying mechanisms. **α**-Mangostin (7.5 **μ**g/mL) activated caspase activity, with a 3-fold and 4-fold increased caspase 8 and 9 activity, respectively. The molecular mechanisms were investigated by qRT-PCR for mRNA related to cell cycle arrest in G_1_ phase (p21^WAF1^ and cyclin D1), apoptosis (cytochrome C, Bcl-2, and Bax), and survival pathways (Akt1, NF**κ**B, and I**κ**B**α**). **α**-Mangostin significantly upregulated mRNA expression of cytochrome C and p21^WAF1^ and downregulated that of cyclin D1, Akt1, and NF**κ**B. **γ**-Mangostin significantly downregulated mRNA expression of Akt1 and NF**κ**B and upregulated p21^WAF1^ and I**κ**B**α**. 8-Deoxygartanin significantly upregulated the mRNA expression of p21^WAF1^ and downregulated that of cyclin D1 and NF**κ**B. The three xanthones significantly inhibited the mRNA expression of the BRAF V600E mutation. Moreover, **α**-mangostin and **γ**-mangostin significantly downregulated Akt phosphorylation at Ser473. In conclusion, the three xanthones induced an inhibitory effect on SK-MEL-28 cells by modulating the molecular targets involved in the apoptotic pathways.

## 1. Introduction

Melanoma, the most fatal form of skin cancer, has been increasing in incidence steadily worldwide for decades, especially in populations of fair-skinned Caucasians [1, 2]. Although early stage melanoma is effectively removed by surgery, later stages of this disease are difficult to treat and have a low survival rate. Chemotherapies (e.g., Dacarbazine) are used to treat advanced melanoma. However, their therapeutic efficacy is limited due to chemoresistance and toxicity issues [3]. Therefore, new agents with a higher therapeutic efficacy are needed for human melanoma.

Xanthones, a group of naturally occurring phenolic compounds, are plentiful in the pericarp of mangosteen (*Garcinia mangostana* Linn.) [4] and are also found in other plants [5]. Anticancer activity is an important biological activity of xanthones. Some xanthones have been shown to inhibit the proliferation of a range of human cancer cells, as described previously [6–8]. Also, *α*-mangostin, a major xanthone compound, can inhibit the metastasis of some types of cancer cells *in vitro* and *in vivo* [7]. Our group demonstrated the potent cytotoxicity of three xanthones (*α*-mangostin, *γ*-mangostin, and 8-deoxygartanin) isolated from mangosteen pericarp against human melanoma SK-MEL-28 cells [9]. Among these, *α*-mangostin showed the strongest activity. We demonstrated that the effect of the three xanthones was associated with cell cycle arrest in G_1_ and induction of apoptosis via caspase 3 activation and mitochondrial membrane disruption. However, the molecular mechanism by which these xanthones exert growth regulatory and apoptotic effects was not known. Therefore, the current study determined the underlying mechanisms of xanthone-induced cell cycle arrest and apoptosis by investigating the expression of genes potentially involved.

In many cancers, activation of antiapoptotic factors can lead to chemoresistance, as can reinforcement of survival pathways. In melanoma, the PI3K/Akt (Akt) signalling pathway is constitutively activated through multiple mechanisms. Activated Akt can phosphorylate many downstream targets (e.g., Bad, Bax, and caspase-9) and suppress proapoptotic transcription factors (e.g., FoxO and p53) [10–13]. Consequently, activation of this signalling pathway can block apoptosis, and thus supporting cancer cell growth [14, 15]. Also, activation of NF*κ*B has been observed in melanoma [16] and has been associated with resistance to radiotherapy and chemotherapy [17–19]. Xanthones may inhibit melanoma cell proliferation via regulation of these signalling pathways. Therefore, the current study examined the effect of downregulation of gene transcription in the survival pathways of Akt1 and NF*κ*B, in addition to a number of genes related to apoptosis and cell cycle progression.

## 2. Materials and Methods

### 2.1. Materials

*α*-Mangostin and 8-deoxygartanin were from Phenomenex Australia Pty Ltd. (NSW, Australia) and *γ*-mangostin was from Biopurity Phytochemicals (Chengdu, China) at greater than 98% purity. Trypsin-EDTA and trypan blue staining were from Sigma-Aldrich (St. Louis, USA). Chemicals and xanthone preparation were as described in Wang et al. [9].

### 2.2. Cell Culture and Cell Treatment

Human melanoma SK-MEL-28 cell line was from the American Type Culture Collection (ATCC HTB-72). Cells were cultured in DMEM (Sigma, USA) supplemented with 10% heat-inactivated fetal bovine serum (FBS; Invitrogen Corporation, Australia), 100 U/mL penicillin, and 0.1 mg/mL streptomycin (Thermo Scientific, Melbourne, Australia). Cells were maintained in an incubator with 5% CO_2_ at 37°C. Cells were free of mycoplasma contamination as detected by PCR (data not shown). Cell treatment was for 48 h as described in Wang et al. [9].

### 2.3. Caspase-8 and 9 Assay

Caspase activity was measured by using Caspase-Glo 8 and 9 assay kits according to the manufacturer's instructions (Promega Corporation, Australia) [8]. 

### 2.4. Apoptosis Assay

To confirm the role of caspase in the apoptosis induced by these xanthones, pan-caspase inhibitor (Z-VAD-FMK) (Promega Corporation, Australia) was used. The inhibitor (60 *μ*M) was added 2 h before the addition of the xanthone. The apoptosis induced by xanthone alone or xanthone with caspase inhibitor (CI) was determined using Annexin V-FITC and PI staining detected by flow cytometry as previously described [9].

### 2.5. Cytochrome C Release Measurement

The release of cytochrome C from the mitochondrial membrane was detected using the InnoCyte Flow Cytometric Cytochrome C Release Kit according to the manufacturer's instructions (Merck, Australia). Briefly, SK-MEL-28 cells were harvested after 48 h treatment with the tested xanthones. 10^6^ cells were resuspended in 300 *μ*L Permeabilization Buffer and incubated for 10 min on ice. Then the cells were fixed with 4% paraformaldehyde and incubated for 20 min at room temperature, followed by being washed for three times using 1× washing buffer. The cells were incubated with blocking buffer for 1 h at room temperature. After that, the cells were incubated with anticytochrome C mouse monoclonal antibody, followed by secondary antibody conjugated to FITC. Finally, the cells were resuspended in 500 *μ*L of washing buffer and analysed by FACS Calibur Flow Cytometer (Becton Dickinson, San Jose, CA, USA).

### 2.6. qRT-PCR

Real time reverse transcription-PCR (qRT-PCR) was performed as described previously [8] to determine mRNA levels of a number of cell cycle-related genes (p21^WAF1^ and cyclin D1), apoptosis-related genes (Bcl-2, Bax, and cytochrome C), and prosurvival-related genes (Akt, NF*κ*B, I*κ*B*α*, and BRAF V600E) on the SK-MEL-28 cells following the 48 h treatments. The details of primer sequences are contained in Wang et al. [8] with the addition of BRAF V600E primer set (F: AGGTGATTTTGGTCTAGCTACAGA; R: TAGTAACTCAGCAGCATCTCAGGGC; Accession number: HM459603.1; Product size: 149 bp).

### 2.7. Western Blotting Analysis

Protein was isolated using TRIzol solution (Invitrogen, Australia) according to the manufacturer's instructions. Protein concentration was determined using an EZQ protein quantitation kit (Molecular Probes) using bovine serum albumin as the standard. The total of 20 *μ*g of each sample was mixed with sodium dodecyl sulphate (SDS) sample buffer (50 mM Tris-HCl pH 6.8, 4% (v/v) glycerol, 0.8% (w/v) SDS, and 40 mM DTT) and subjected to SDS-PAGE using Criterion TGX Stain-Free precast gel (Bio-Rad) as described previously [20]. Total protein was imaged before and after transfer using a ChemiDoc MP imager (Bio-Rad). Proteins were transferred to Immun-Blot LF polyvinylidene difluoride (PVDF) membrane (0.45 *μ*m; Bio-Rad) using a Turbo Blot transfer unit (Bio-Rad). The membrane was then blocked with 5% (w/v) nonfat dry skim milk in TBS-T (20 mM Tris base, 150 mM NaCl, 0.1% (v/v) Tween-20, pH 7.4) for 1 h at room temperature, followed by overnight incubation with primary antibody at 4°C on a shaker. Nonbinding primary antibodies were removed by 2 × 5 min and 2 × 10 min washes in TBS-T prior to incubation with secondary antibodies (1 : 5000 dilution) for 1 h at room temperature. The membrane was then washed (2 × 5 min and 2 × 10 min washes in TBS-T) and placed in equal parts of stable peroxide buffer and luminol/enhancer solutions of SuperSignal West Pico Chemiluminescent Substrate (Thermo Scientific, Illinois, USA) for 5 min. Enhanced chemiluminescence was detected using a LAS-4000 imager (FujiFilm Global, Tokyo, Japan). All images were analysed using Image J software to estimate the optical densities for each protein band. To account for differences in loading, the net intensity of the band of interest from each sample was normalised to the net intensity of total protein within the same lane. Changes in abundance of the protein of interest due to treatment were compared by normalising to the untreated cells control. 

### 2.8. Statistical Analysis

All experiments were repeated at least three times independently. Means and standard error of mean (SEM) were calculated using Microsoft Excel 2007. The statistical significance of the results was analysed using one-way ANOVA followed by Tukey's HSD *post hoc *test (equal variances) or Dunnett's T3 *post hoc *test (unequal variances). The analysis was carried out using SPSS software (version 18). *P* < 0.05 was considered statistically significant and *P* < 0.01 as highly significant.

## 3. Results

### 3.1. *α*-Mangostin Increased Caspase 8 and 9 Activity of SK-MEL-28 Cells

**Figure 1:**
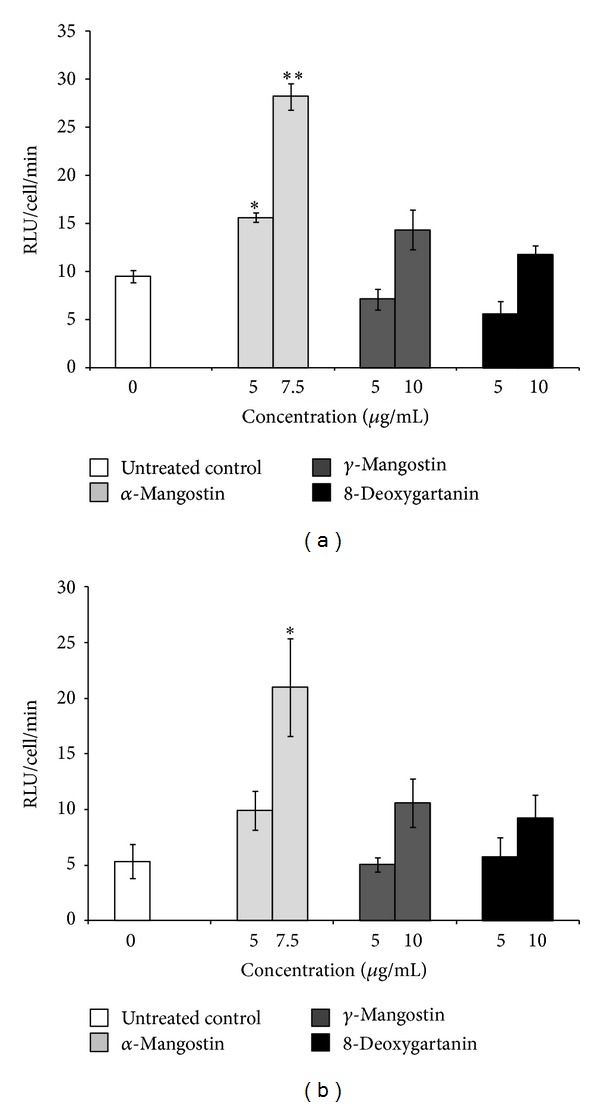
Caspase 8 (a) and 9 (b) activities were determined using luminescent kits as described in the method for SK-MEL-28 cell line treated with xanthones for 48 h. The values are shown as the mean ± SEM (*n* = 3). Treatments significantly different from the untreated control at *P* < 0.05 are presented as ∗ and at *P* < 0.01 as ∗∗.

A significant increase in caspase 8 after 48 h treatment of SK-MEL-28 cells was observed only with *α*-mangostin. Treatment with *α*-mangostin at 5 *μ*g/mL resulted in an approximately 1.6-fold increase (*P* < 0.05) and at 7.5 *μ*g/mL resulted in an approximately 3-fold increase (*P* < 0.01) relative to untreated cells (Figure 1(a)).

A significant increase in caspase 9 was also observed after 48 h treatment with *α*-mangostin only. Treatment with *α*-mangostin at 7.5 *μ*g/mL resulted in an approximately 4-fold increase (*P* < 0.05) relative to untreated cells (Figure 1(b)).

### 3.2. Apoptosis Induced by *α*-Mangostin in SK-MEL-28 Cells Was Rescued by Pan-Caspase Inhibitor

**Figure 2:**
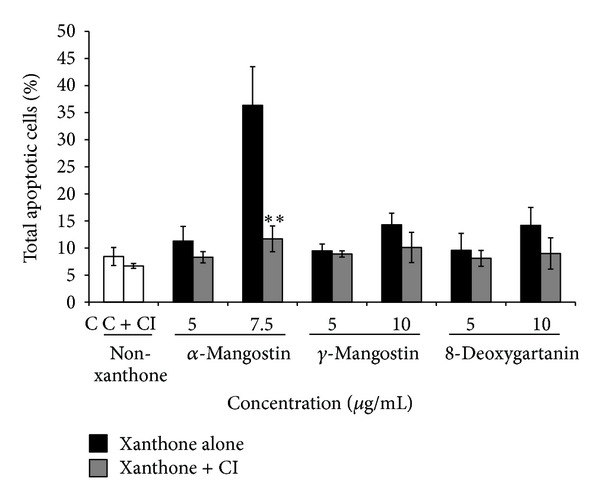
The total of apoptotic cells was determined using Annexin V-FITC and PI staining after treatment with xanthone or with combination of xanthone and caspase inhibitor (CI). The values are shown as the mean ± SEM (*n* = 3). Significant difference in the total apoptotic cells between xanthone alone and combination of xanthone and CI is presented as ∗ (*P* < 0.05) and ∗∗ (*P* < 0.01). C stands for untreated control.

The role of caspase in the apoptosis induced by xanthones was confirmed using pan-caspase inhibitor. As shown in Figure 2, the *α*-mangostin-induced apoptosis was reduced significantly when the pan-caspase inhibitor was applied. The percentage of apoptotic cells decreased to 11.7% in the presence of the inhibitor from 36.4% in the absence of the inhibitor (Figure 2). However, no significant difference was observed after treatment with *γ*-mangostin and 8-deoxygartanin.

### 3.3. Xanthones Increased Cytochrome C Release from Mitochondria to Cytosol

**Figure 3:**
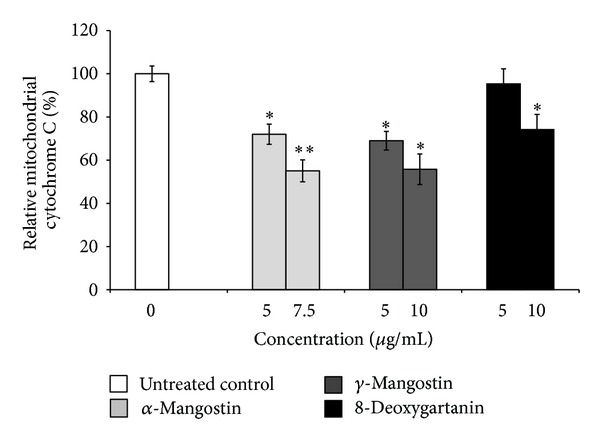
Mitochondrial cytochrome C was examined using flow cytometry after 48 h treatment with *α*-mangostin, *γ*-mangostin, and 8-deoxygartanin. Data were obtained from 20,000 events and presented as the percentage of mitochondrial cytochrome C compared to the untreated control. The values are shown as the mean ± SEM of three independent experiments. Treatments significantly different from the untreated control at *P* < 0.05 are presented as ∗ and *P* < 0.01 as ∗∗.

Increases in cytochrome C release from mitochondria to cytosol were observed for all treatments. Treatment of SK-MEL-28 cells with **α**-mangostin, *γ*-mangostin, and 8-deoxygartanin significantly decreased % mitochondrial cytochrome C to 55.1%, 55.8%, and 74.1% compared to the untreated cells (100%), respectively (Figure 3).

### 3.4. Xanthones Modulated Cell Cycle-Related Gene Expression

Our previous study demonstrated that treatment with xanthones induced significant increases in cell cycle arrest in G_1_ phase in SK-MEL-28 cells [9]. The current study examined the effect of these xanthones on the modulation of the cell cycle-related gene expression in SK-MEL-28 cells.

**Figure 4:**
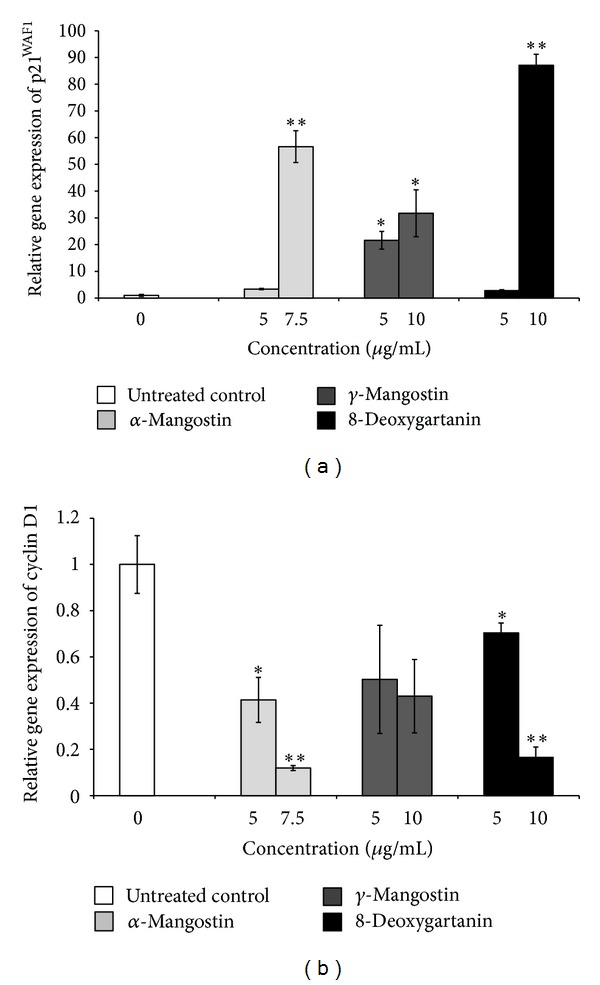
Effect of xanthones on the mRNA expression of (a) p21^WAF1^ and (b) cyclin D1 as determined by qRT-PCR for SK-MEL-28 cell line treated with xanthones for 48 h. The values are shown as the mean ± SEM (*n* = 4). Treatments significantly different from the untreated control at *P* < 0.05 are presented as ∗ and at *P* < 0.01 as ∗∗.

Significant increases in mRNA level of p21^WAF1^ were observed after treatment with the three tested compounds (Figure 4(a)). The most marked effect was for 8-deoxygartanin, which induced an 87.1-fold increase of the mRNA level of p21^WAF1^ relative to the untreated control (*P* < 0.01). 

Significant decreases in the mRNA level of cyclin D1 were observed after treatment with *α*-mangostin and 8-deoxygartanin (Figure 4(b)). A 10-fold and 5-fold decrease of the mRNA level of cyclin D1 relative to the untreated control was induced by *α*-mangostin (*P* < 0.01) and 8-deoxygartanin (*P* < 0.01), respectively.

### 3.5. Xanthones Modulated Apoptosis-Related Genes

**Figure 5:**
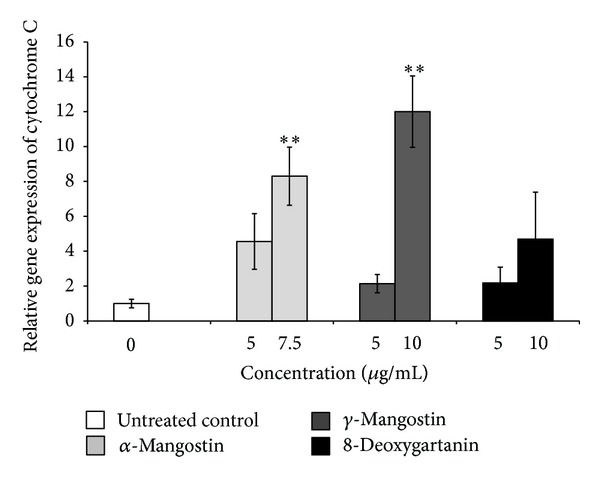
Effect of xanthones on the mRNA expression of cytochrome C as determined by qRT-PCR for SK-MEL-28 cell line treated with xanthones for 48 h. The values are shown as the mean ± SEM (*n* = 5 for *γ*-mangostin and *n* = 3 for *α*-mangostin and 8-deoxygartanin). Treatments significantly different from the untreated control at *P* < 0.05 are presented as ∗ and at *P* < 0.01 as ∗∗.

To understand the molecular mechanisms of apoptosis induced by xanthones, we firstly examined the effect of xanthones on the mRNA expression of cytochrome C, which is an important signalling event in the intrinsic apoptotic activation pathway. The significant increases found after 48 h treatment with *α*-mangostin (7.5 *μ*g/mL) and *γ*-mangostin (10 *μ*g/mL) (Figure 5) were 8-fold and 12-fold, respectively.

We also examined the effect of xanthones on the mRNA level of Bax and Bcl-2. However, no significant alterations in the Bax and Bcl-2 were found after treatment with the tested xanthones (data not shown).

### 3.6. Xanthones Modulated Genes in Survival Pathways

**Figure 6:**
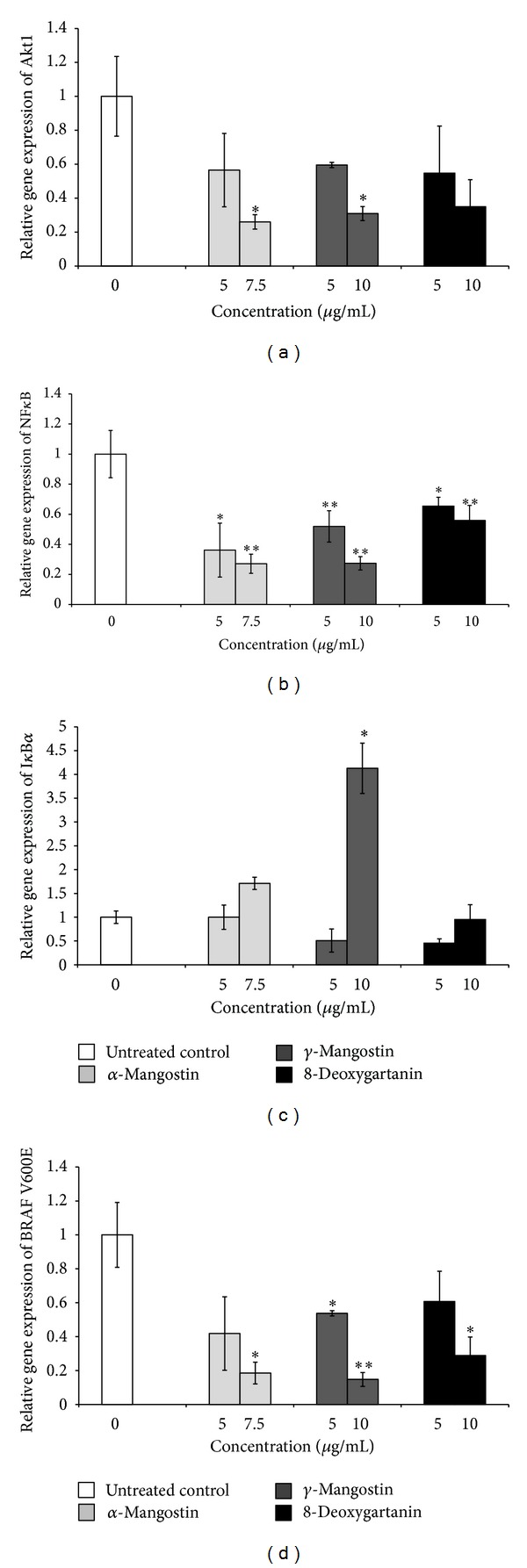
Effect of xanthones on the mRNA expression of (a) Akt1, (b) NF*κ*B, (c) I*κ*B*α*, and (d) BRAF V600E mutation as determined by qRT-PCR for SK-MEL-28 cell line treated with xanthones for 48 h. The values are shown as the mean ± SEM (*n* = 3). Treatments significantly different from the untreated control at *P* < 0.05 are presented as ∗ and at *P* < 0.01 as ∗∗.

Treatment of SK-MEL-28 cells with *α*-mangostin (7.5 *μ*g/mL) and *γ*-mangostin (10 *μ*g/mL) induced 3.8- and 3.2-fold decreases of the mRNA level of Akt1, respectively (*P* < 0.05; Figure 6(a)).

Significant decreases of the mRNA level of NF*κ*B were found for SK-MEL-28 cells after treatment with 7.5 *μ*g/mL of *α*-mangostin (3.7-fold), 10 *μ*g/mL of *γ*-mangostin (3.7-fold), and 10 *μ*g/mL of 8-deoxygartanin (1.7-fold) (Figure 6(b)).

However, a significant increase of the mRNA level of I*κ*B*α* was found only after treatment with one concentration of *γ*-mangostin (10 *μ*g/mL) with a 4.1-fold increase relative to the untreated control (*P* < 0.05; Figure 6(c)).

Significant decreases in the mRNA level from the BRAF V600E mutant gene were observed after treatment with *α*-mangostin, *γ*-mangostin, and 8-deoxygartanin (Figure 6(d)). The most marked effect was with *γ*-mangostin, which induced a 6.8-fold decrease of the mRNA level of BRAF V600E relative to the untreated control (*P* < 0.01).

### 3.7. Xanthones Modulated Protein Expression of Akt1 and Phosphorylation at Ser473 and Thr308

**Figure 7:**
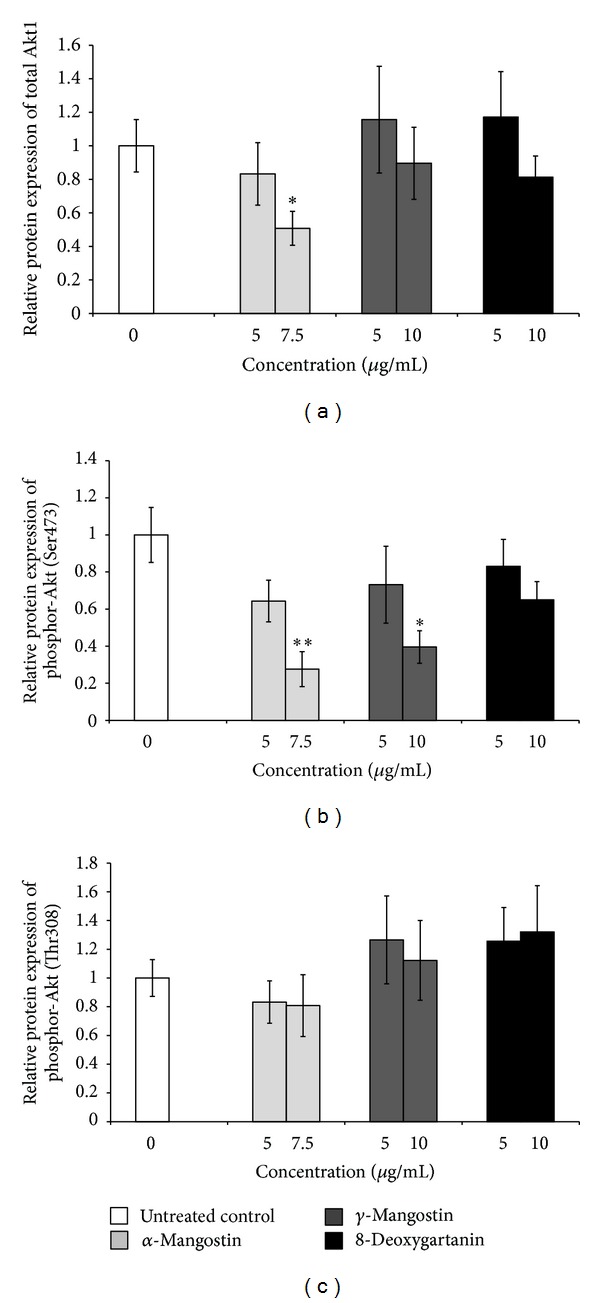
Effect of xanthones on the protein expression of Akt1 (a), phosphor-Akt (Ser473) (b), and phosphor-Akt (Thr308) (c) in SK-MEL-28 cell line was determined by western blot analysis. The values are shown as the mean ± SEM of three independent experiments. Treatments significantly different from the untreated control at *P* < 0.05 are presented as ∗ and at *P* < 0.01 as ∗∗.

*α*-Mangostin significantly inhibited the protein expression of Akt1 and phosphor-Akt (Ser473) in SK-MEL-28 cells (Figures 7(a) and 7(b)). *γ*-Mangostin inhibited the expression of phosphor-Akt (Ser473) (Figure 7(b)). No significant changes in phosphor-Akt (Thr308) were found after treatment with the tested three xanthones (Figure 7(c)).

## 4. Discussion and Conclusion

In the present study, we investigated the potential mechanisms underlying the antiproliferative effect of the three xanthones on human melanoma SK-MEL-28 cells. Our previous study demonstrated that treatment of SK-MEL-28 cells with xanthones induced G_1_ phase arrest [9]. The G_1_ phase of cell cycle is controlled by cyclin dependent kinases, cyclin kinase inhibitors (CKI), and cyclins [21]. qRT-PCR was used to measure the mRNA expression levels of genes involved in cell cycle regulation. The genes studied were cyclin D1 (involved at the G_1_/S boundary) and p21^WAF1^ (a universal CKI). The expression of p21^WAF1^ was significantly increased after treatment of SK-MEL-28 cells with the three xanthone compounds. SK-MEL-28 cells possess mutant p53 [22], therefore the xanthone-induced increases in p21^WAF1^ could be p53 independent. Additionally, significant decreases in the expression of cyclin D1 were observed after treatment with *α*-mangostin and 8-deoxygartanin. Consistently, on human prostate cancer 22Rv1 cells, *α*-mangostin has been reported to induce cell cycle arrest in G_1_ phase by inhibition of CDK4 via upregulating protein expression of p27^Kip1^ and downregulating protein expression of cyclin D1 and D3, phosphorylated retinoblastoma, and cyclin E [23]. The mechanism of cell cycle arrest induced by xanthones could be studied further, for example, via the expression of additional cyclins (e.g., A/E) and cyclin-dependent kinases (CDK) (e.g., CDK2/4/6).

Apoptosis plays a critical role in the prevention of cancer. However, most cancer cells show resistance to cell death, and melanoma cells are no exception. The resistance to apoptosis could be required for tumour growth and likely contributes to chemoresistance [20]. The resistance of melanoma to apoptosis may be due to inactivation of proapoptotic effectors (e.g., loss of the p53 pathway) [24, 25] and activation of anti-apoptotic factors (e.g., Bcl-2, Bcl-xL, and Mcl-1 are highly expressed in melanoma cells) [26–28]. Also, melanoma shows resistance to death receptor-mediated apoptosis (extrinsic pathway), possibly due to resistance to CD95 stimulation [29], and resistance to TNF-*α*-mediated apoptosis [30]. We previously demonstrated that the three tested xanthones, especially *α*-mangostin, induced apoptosis on human SK-MEL-28 cell line [9]. The mechanisms of apoptosis are highly complex, involving multiple molecular events. Generally, there are two major apoptotic pathways: the intrinsic or mitochondrial pathway and the extrinsic or death receptor pathway [31, 32]. Caspases 3 and 7 are effector caspases which are involved in both pathways. Caspases 8 and 9 are initiator caspases which are involved in the extrinsic and the intrinsic pathways, respectively [33, 34]. Both *α*-mangostin and 8-deoxygartanin activated caspase 3/7 in human melanoma SK-MEL-28 cells [9]. The current study demonstrated that only *α*-mangostin induced significant activation of caspases 8 and 9 (Figures 1(a) and 1(b)). Consistently, *α*-mangostin previously increased caspases 3, 8, and 9 activities in human chondrosarcoma SW1353 cell line [35]. However, this is not in agreement with findings of Matsumoto et al. [36] who reported that *α*-mangostin activated caspases 3 and 9, but not caspase 8. This apparent conflict in the data may be because this compound exerts its activities via different pathways in different types of cancer cells [7]. After treatment with 8-deoxygartanin, caspase 9 activity was only slightly increased, which was not significant. The results indicated that *α*-mangostin could induce apoptosis through both intrinsic and extrinsic pathways. Previously, the three tested xanthones significantly decreased the mitochondrial membrane potential of SK-MEL-28 cells [9], indicating that *γ*-mangostin induced caspase-independent apoptosis via the mitochondrial pathway. Caspase-independent apoptosis via the mitochondrial pathway has also been reported after treatment of colon cancer cells with *α*-mangostin [37]. In this study, activation of caspases was confirmed using caspase inhibitors. Addition of the caspase inhibitor significantly rescued the cell death induced by *α*-mangostin (Figure 2), indicating that *α*-mangostin-induced apoptosis is mediated through the activation of caspases.

To determine the molecular mechanism underlying the apoptosis induced by xanthones, the genes involved in apoptosis were analyzed by qRT-PCR. Mitochondrial membrane potential disruption leads to the release of cytochrome C into the cytoplasm. This will result in promotion of the formation of the apoptosome and activation of caspase-9 (initiator caspase) and consequent activation of downstream caspases 3 and 7 (executioner caspases) [38, 39]. We found that treatment with *α*-mangostin and *γ*-mangostin significantly upregulated the mRNA expression of cytochrome C in SK-MEL-28 cell line. Transcriptional activation of cytochrome C can lead to increased protein expression in both the cytosol and mitochondria [40]. The levels of cytochrome C in the cytosol and mitochondria after treatment were determined by flow cytometry. The results demonstrated increases in cytochrome C release from mitochondria to cytosol, suggesting the engagement of the mitochondria-mediated apoptotic pathway. 

The Bcl-2 family of proteins regulates the integrity of the mitochondrial membrane and the efflux of proapoptotic proteins from the mitochondria. There are three groups in the Bcl-2 family: the first group is antiapoptotic (e.g., Bcl-2 and Bcl-Xl), acting to preserve mitochondrial integrity and prevent cells from apoptosis [41]; the second group is proapoptotic (e.g., Bax and Bak), acting to disrupt the mitochondrial membrane and promote cell apoptosis [42]; and the third group is a large family (e.g., Bim, Bad, Bid, Noxa, and Puma), interacting with other Bcl-2 family members [43]. In this study, we examined the changes of mRNA level in Bcl-2 (anti-apoptotic) and Bax (proapoptotic) after treatment with xanthones. However, no significant changes were found after treatment with the three xanthones. This suggests that these two genes are not involved in the apoptosis induced by the three xanthones. This is consistent with the studies of Matsumoto et al. [36] and Wang et al. [8]. In contrast, Krajarng et al. [35] found that *α*-mangostin increased Bax protein expression and decreased Bcl-2 in human chondrosarcoma SW1353 cell line. Thus, the effect of *α*-mangostin appears to be cancer type dependent. It would be interesting to monitor the protein levels of Bcl-2 and Bax for the three xanthones to clarify the current result.

Akt plays an essential role in controlling cell survival, growth, and apoptosis. It is constitutively activated in many human cancers [44] by phospholipid binding and activation loop phosphorylation at threonine 308 (Thr308) and by phosphorylation within the carboxy terminus at serine 473 (Ser473) [45]. We demonstrated that treatment with *α*-mangostin and *γ*-mangostin significantly downregulated the Akt1 mRNA expression (Figure 6(a)) and the Akt phosphorylation at Ser473 (Figure 7(b)). Downregulation at Thr308 was not observed. However, Shibata et al. [46] reported that *α*-mangostin suppressed phospho-Akt-Thr308 but not Ser473 in human mammary carcinoma MDA-MB231 cells. Also, *α*-mangostin decreased the phosphorylation of Akt in chondrosarcoma cells without affecting the total Akt protein [35]. This difference might be due to the cell type investigated. Further study is required to clarify the different responses of different cell types to xanthone compounds.

NF*κ*B is involved in regulation of cellular differentiation, proliferation, and apoptosis. It contains five members with p65 and p50 being the most abundant ones. All three tested xanthones significantly downregulated the mRNA expression of NF*κ*B on the SK-MEL-28 cells. Consistently, *α*-mangostin and 8-deoxygartanin inhibited p65 activation with IC_50_ values of 15.9 and 3.2 *μ*M, respectively, in an ELISA NF*κ*B assay [47]. I*κ*B*α* plays a key role in the NF*κ*B pathway. Phosphorylation of I*κ*B*α* by IKK and subsequent degradation is required for the activation of NF*κ*B [16]. In the current study, a significant increase in the mRNA level of I*κ*B*α* was found after treatment with *γ*-mangostin on SK-MEL-28 cells. These results support a role for I*κ*B*α* in the inhibitory effect of NF*κ*B induced by *γ*-mangostin on SK-MEL-28 cells. ERK is another important upstream regulator for NF*κ*B. Therefore, the ERK pathway might be involved in downregulation of NF*κ*B expression induced by the xanthones tested. Thus, the mRNA and protein levels of these molecular targets warrants further investigation.

BRAF gene mutations have been reported in most malignant melanomas [48, 49]. BRAF V600E mutation accounts for 89-90% of all the detected BRAF mutations. BRAF V600E mutation leads to constitutive activation of the MEK/ERK pathway, promoting cancer cell survival and proliferation [50]. In the current study, significant downregulation of mRNA expression of BRAF V600E was observed for human melanoma SK-MEL-28 cells treated with *α*-mangostin, *γ*-mangostin, and 8-deoxygartanin (Figure 6(d)). RG7204 (PLX4032), a selective BRAF V600E inhibitor, has potent inhibitory effects on the growth of melanoma harboring this mutation both *in vitro* and *in vivo* [51]. Particularly, PLX4032 was reported to inhibit the growth of SK-MEL-28 cells [52]. It is currently in phase II and phase III clinical trials [51]. GSK2118436 is another successful example of treating melanoma by targeting BRAF mutations [53]. The current results indicate that xanthones have potential as antimelanoma drugs via targeting of the BRAF V600E mutation. Given the role of BRAF V600E in the MAPK pathway, it is predicted that *α*-mangostin could inhibit the activation of MEK/ERK. Consistently, *α*-mangostin has been reported to downregulate MAPK pathway by inhibiting ERK and JNK on human colon DLD-1 cells [54] and human chondrosarcoma SW1353 cells [35]. To the best of our knowledge, this study is the first demonstration of the inhibitory effect of xanthone compounds on BRAF V600E mutation. It would be interesting to investigate the effect of xanthones on the activation of MEK/ERK in SK-MEL-28 cells using western blotting analysis in the future. 

In the current study, the gene expression was tested at 48 h when significant apoptosis occurred. However, if the target gene expression was only examined at this one time point, the alteration of mRNA expression induced by xanthone compounds may be underestimated. The changes at transcriptional level occur earlier than those at protein level. Before the apoptosis is manifest at the cellular level, the mRNA expression level of target genes may reach a peak and then decrease. In the future, additional time points early in the process of apoptosis could be studied (e.g., 2, 4, 6, 12, and 24 h) to detect the peak of mRNA expression of the target genes. Additionally, the effect of xanthones could be tested on a range of melanoma cell lines with different genetic backgrounds to investigate the role of mutant genes in influencing efficacy. Selectivity of xanthones should also be tested using noncancerous melanocyte cells in the future. Moreover, the other molecular targets in this study need to be verified at the protein level by Western Blot analysis. *In vivo* studies are required to determine the therapeutic efficacy of xanthones.

In conclusion, our study indicates that the antiproliferative and apoptotic effects of these three xanthones on human melanoma SK-MEL-28 cell line are via modulation of the genes involved in the cell cycle and apoptosis pathway, together with downregulation of the mRNA expression of Akt1, NF*κ*B, and BRAF V600E. Understanding mechanisms of xanthone action should allow for the design of combination treatment using xanthones with other therapies, based on molecular targets.
